# Visualization of Local Concentration and Viscosity Distribution during Glycerol-Water Mixing in a Y-Shape Minichannel: A Proof-of-Concept-Study

**DOI:** 10.3390/mi12080940

**Published:** 2021-08-10

**Authors:** Isabel Medina, Julian Deuerling, Pooja Kumari, Stephan Scholl, Matthias Rädle

**Affiliations:** 1 Center for Mass Spectrometry and Optical Spectroscopy, Mannheim University of Applied Sciences, Paul-Wittsack-Straße 10, 68163 Mannheim, Germany; i.medina@hs-mannheim.de (I.M.); j.deuerling@hs-mannheim.de (J.D.); p.kumari@hs-mannheim.de (P.K.); 2 Technische Universität Braunschweig, Institute for Chemical and Thermal Process Engineering, Langer Kamp 7, 38106 Braunschweig, Germany; s.scholl@tu-braunschweig.de

**Keywords:** concentration-distribution imaging, glycerol, microchannel, near-infrared, viscosity, water

## Abstract

The work presents an efficient and non-invasive method to visualize the local concentration and viscosity distribution of two miscible and non-reacting substances with a significant viscosity difference in a microchannel with a Y-shape cell. The proof-of-concept setup consists of a near-infrared (NIR) camera and cost-effective dome lighting with NIR light-emitting diodes (LED) covering the wavelength range of 1050 to 1650 nm. Absorption differences of glycerol and water and their mixtures with a mass fraction of glycerol from 0 to 0.95 $g_{Glyc}g_{total}^{-1}$ were analyzed in the NIR spectral area. The resulting measurement images were converted in a concentration profile by using absorbance calculated with Lambert–Beer law. A linear behavior between the concentration and the absorption coefficient is demonstrated. The result of local concentration in mass fraction was used to determine the local viscosity and illustrated as distribution images. By variating the fluid parameters, the influences of the highly different original viscosities in the mixing procedure were investigated and visualized.

## 1. Introduction

During the last few years, the application of micro-structured components for process engineering has gained increasing importance in chemical, pharmaceutical, and life sciences. These components vary in size, but all devices can be fabricated in configurations scaled in millimeters and embedded with micrometer-sized channels. These devices can be reactors, heat exchangers, and static mixers, among other process components [1]. An extremely high surface-to-volume ratio characterizes microchannel-based devices because of their small linear dimensions. Due to this property, they gain most of their advantages over conventional-sized chemical process equipment [2]. It results in more efficiency for chemical reactions and mass and heat transfer [3]. Additionally, fluids in channels of micro-scale dimensions behave differently as compared to macroscopic geometries. The analysis of mixing efficiency in microchannels consists of determining crucial parameters, in this case, the local concentration and viscosities [4,5,6]. In this study, concentration profiles and an indirect calculation of viscosity were investigated by using a new approach of near-infrared imaging with an optical measurement method to evaluate the mixing process of fluids with very different properties, such as water and glycerol, in a cell with a Y shape and by using the Lambert–Beer law for comprehensive absorbance analyses. A Y-shaped mini-channel was selected to verify the measurement using a basic geometry with a distinct observation of the fluids in the inlet of the channel.

Currently, there are various inline measurement methods for microreactor analysis, including spectroscopic methods. Raman is widely used in studies for diffusion and interdiffusion processes in microchannels [7,8]. Raman spectroscopy was also used for interdiffusion analysis with liquids, such as a water and glycerol-water mixture, with different viscosities [9] or for monitoring the hydrolysis of acetal in microreactors [3]. The performance of mixing of viscoelastic fluids in a microchannel and convective mixing were analyzed with fluorescence imaging [10,11], and complex concentration profiles were also examined with a fluorescence microscope [12]. The dynamics of glycerol-water mixtures have also been separately analyzed with numerical methods [13] and with broadband dielectric spectroscopy (BDS) and differential scanning calorimetry (DSC) [14]. However, these methods differ from the method presented in this work and have several disadvantages, like the dependency on tracer substances and slow measurement speed. The novelty of the work is a fast, non-invasive measurement technique and the detection of the concentration and gaining information of viscosity in a large area at the same time. Therefore, near-infrared spectroscopy proves to be a promising measurement technique and has been used to monitor processes [15] and control the quality of many products, such as biodiesel, by identifying the traces of water and glycerol or water drops in oil [16,17]. Other applications involve measuring film thickness, concentration, or temperature of the water or aqueous solutions [18,19,20,21]. Glycerol has proven to be an appropriate substance in experiments where viscosity plays an important role and is widely used in scientific and industrial fields, and its use is well suited to the analysis of viscous solutions. It requires little effort because the properties, like uncomplex solubility, non-toxicity, low cost, and high viscosity, alleviate the experimental complications for the application [22]. 

This work aimed to experimentally determine the local concentration of glycerol-water mixtures using near-infrared imaging in a mini-channel structure and an indirect calculation of local viscosity. Experimental efforts contributed to investigations of two-dimensional mapping in mini-channels.

## 2. Materials and Methods 

### 2.1. Concentration Analysis 

The analysis of the characteristics of the water-glycerol mixtures has been conducted for many years. In the literature, piles of data are available, measured at different temperatures [23,24,25,26,27]. Cheng [28] proposed a formula to calculate the dynamic viscosity of a glycerol-water mixture for a temperature range from 0 to 100 °C:

(1) $$\eta_{mix}=\eta_{w}^{\alpha }\eta_{g}^{1-\alpha }$$

The dynamic viscosities of pure water $\eta_{g}$ and glycerol $\eta_{w}$ depend on the temperature ϑ and are formulated as

(2) $$\eta_{w}=1.790exp\left(\frac{\left(-1230-\vartheta \right)\vartheta }{36,100+360\vartheta }\right)$$

(3) $$\eta_{g}=12,100exp\left(\frac{\left(-1233+\vartheta \right)\vartheta }{9900+70\vartheta }\right)$$

The calculated dynamic viscosities are in mPa s and the temperature in °C. For the dynamic viscosity calculation of the mixture, the weighting factor *α* from 0 to 1 is given by Equation (4), whereby $x_{Glyc}$ is the mass fraction of glycerol-water mixture:

(4) $$\alpha =1-x_{Glyc}+\frac{abx_{Glyc}\left(1-x_{Glyc}\right)}{ax_{Glyc}+b\left(1-x_{Glyc}\right)}$$

Segur and Oberstar [23] proposed the following relations of a and b as a function of temperature ϑ between 0–100 °C:

(5) a=0.705−0.0017ϑ

(6) $$b=\left(4.9+0.036T\right)a^{2.5}$$

The study is performed with three temperatures: 20, 30, and 40 °C. Density of the mixture and the dynamic viscosity are calculated with the two coefficients, a and b, and shown in Table A1 (Appendix A).

The Reynolds number determines the fluid dynamic state of the flow in the channel and is defined as

(7) $$Re=\frac{\rho u_{m}L}{\eta }=\frac{u_{m}L}{v}$$

Whereby:

- ρ is the density of the fluid $\left(\frac{\mathrm{kg}}{m^{3}}\right)$;
- $u_{m}$ is the velocity of the fluid $\left(\frac{m}{s}\right);$
- L is a characteristic linear dimension (m);
- η is the dynamic viscosity of the fluid $\left(\mathrm{Pa}\;s\;\mathrm{or}\frac{\mathrm{kg}}{m.s}\right)$; and
- v is the kinematic viscosity of the fluid $\left(\frac{m^{2}}{s}\right)$.

For the calculation of the Reynolds number, the density of the water-glycerol mixture is defined by [28]

(8) $$\rho_{mix}=\rho_{g}x_{Glyc}+\rho_{w}\left(1-x_{Glyc}\right)$$

Whereby $x_{Glyc}$ is the mass fraction of glycerol-water mixture; $g_{Glyc}g_{total}^{-1}$, $\rho_{w}$, and $\rho_{g}$ are the densities of water and glycerol for the temperature ϑ between 0–100 °C and are empirically defined by

(9) $$\rho_{w}=1000\left(1-{\left|\frac{\vartheta -4}{622}\right|}^{1.7}\right)$$

(10) $$\rho_{g}=1277-0.654\vartheta$$

In software programming for image analysis, Equations (8) to (10) quantify the viscosity of glycerol-water mixtures based on the concentration analysis and temperature.

### 2.2. Fluid Preparation

The experiment used water and four different glycerol-water mixtures by varying mass fractions of glycerol $x_{Glyc}$ to vary the viscosity. The experiments were carried out with glycerol (ROTIPURAN^®^ ≥ 99.5% p.a., anhydrous, Carl Roth GmbH, Germany) and pure water. The dynamic viscosity of the mixture calculated by Equation (1) is shown in Table 1.

The influence of the temperature is evaluated by pumping water and glycerol in the channel at 20, 30, and 40 °C. Furthermore, the effect of different mixing ratios of water and glycerol was studied and analyzed. 

The dynamic viscosity calculated using Equation (1) was compared with two published experimental measurements [23,25]. The viscosity results using Equation (1) are a suitable approximation to the data measure from Segur and Oberstar [23] and Shankar [25]. The results for mass fraction between 0 and 1 of glycerol-water mixtures are shown in Table A2 (Appendix A) at 20 °C. 

This study implements the calculation using Equation (1) at three temperatures: 20, 30, and 40 °C. The results are approximated with exponential functions, and the full range is separated into two sections: first, from 0 to 0.7 $g_{Glyc}g_{total}^{-1}$ and second, from 0.7 to 1 $g_{Glyc}g_{total}^{-1}$. The fitting of two sections was calculated for adequate analysis.

**Figure 1:**
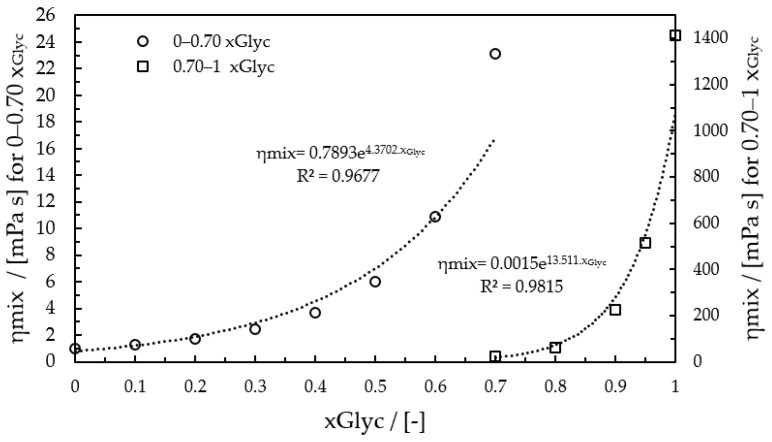
Dynamic viscosity with Equation (1) at 20 °C for the imaging analysis Software.

At 20 °C, the mixture viscosity for *x_Glyc_* ≤ 0.7 $g_{Glyc}g_{total}^{-1}$ is determined by *η_mix_* = $0.7893e^{4.37x}$ with $R^{2}$= 0.9677 and for *x_Glyc_* > 0.7 $g_{Glyc}g_{total}^{-1}$ by *η_mix_* = $0.0015e^{13.51x}$ with $R^{2}$= 0.9815. Figure 1 shows the calculation as a diagram at 20 °C. 

For 30 °C and 40 °C, the equations were calculated and used for imaging analysis. Table 2 resumes the equations for viscosity calculation for the three temperatures.

### 2.3. Spectroscopy

Near-infrared spectroscopy is based on a specific segment in the wavelength range of the electromagnetic spectrum and is suitable to analyze molecular vibrations. In this study, the analysis was performed by using the near-infrared range (NIR and SWIR) from 750 nm to 2500 nm.

The near-infrared spectroscopy detects overtones of structural vibrations, such as –NH, -OH, and –CH as well as combination vibrations, resulting in difficulties in the identification of a substance. In the spectral near-infrared range, the molecular vibration for water is strong. The associated vibration bond is located between 1450 nm and 1460 nm. Glycerol has an overtone between 1460 nm and 1600 nm from the vibration bond of the –OH group and between 1615 nm and 1640 nm from the vibration bond of the –CH group [29]. 

The absorption of light through the material also depends on the molar concentration and the thickness of the substance. This principle facilitates the measurement of the concentration of mixtures through light absorption. The Lambert–Beer law describes the absorption in terms of intensity, as shown in Equation (11). The absorbance $A_{\lambda }$ is defined by the logarithm of the transmission of the intensity of the reference $I_{0}$, in this case, by air, to the intensity after the absorption I depending on the temperature *T*. *λ* is the wavelength of light between 900 nm and 1700 nm. The absorbance is also defined by the extinction coefficient ε, the molar concentration of absorber c, and the layer thickness d.

(11) $$A_{\lambda }=log_{10}\left(\frac{I_{0}\left(\lambda ,T\right)}{I\;\left(\lambda ,T\right)}\right)=\varepsilon \left(\lambda \right)cd$$

This study is based on two main measurement methods: the transmission and remission principles. In transmission measurement, light penetrates the substrate only once and is detected on the other side. In a remission measurement, light penetrates the fluid and is diffusely reflected from a surface. This principle is used for the measurements with the channel in these experiments.

**Figure 2:**
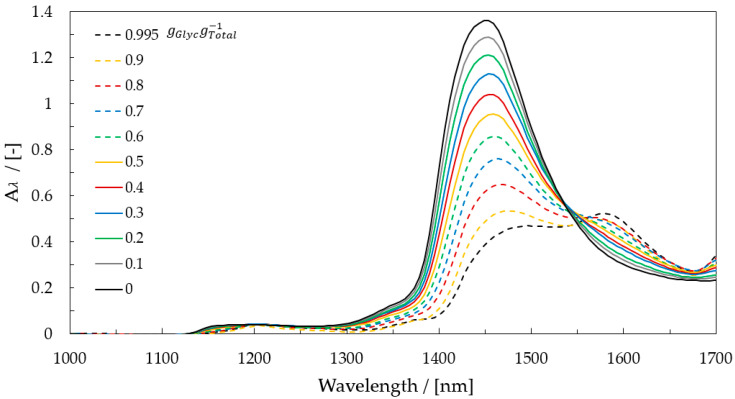
Pure water, glycerol, and glycerol-water mixtures in near-infrared spectra.

The near-infrared spectra of water and glycerol and their mixtures are measured with the transmission measurement principle by a VIS-NIR spectrometer (MCS611 NIR 2.2µ with a halogen lamp CLD600, ZEISS) in a 1-mm path-length cell. The absorbance $A_{\lambda }$, depending on wavelength and temperature, is defined in Equation (11) and is calculated with air as a reference $I_{0}$ (see Figure 2). 

### 2.4. Experimental Setup

An essential part of imaging analysis is the selection of proper lighting. Parameters like homogeneity and intensity define the quality of the pictures and the results of the measurement. The imaging system is based on the dome lightning principle presented in [30]. Light is reflected from the surface of the dome and goes to the object in a diffuse form. Thus, complex reflective objects and fluid shapes can be analyzed. Creating a dome lighting system by using a hollow, hemispherical dome as a reflective chamber and positioning the LEDs onto the sides facilitates a suitable imaging system. 

**Figure 3:**
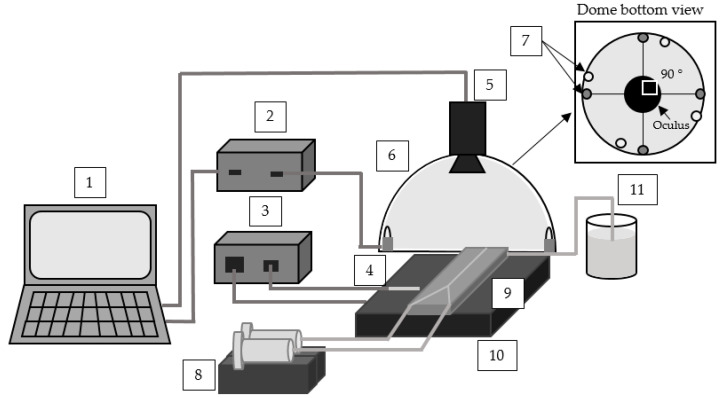
Schematic overview of the experimental setup: computer unit with acquisition software #1, controlling device with microcontroller #2, temperature controller with a temperature sensor type K #3–4, near-infrared camera Goldeye #5, dome lighting #6, near-infrared LEDs, dome (bottom view) #7, syringe pump #8, mini-channel #9, heater #10, and waste container #11.

The measurement system is depicted in Figure 3, and it consists of a dome, which is made of polymer polypropylene, with a diameter of 35 cm and a height of 17 cm with a covering layer of white lacquer that improves the diffusion of the light. Near-infrared-LEDs were placed on the edge of the dome (#6 in Figure 3). For each wavelength, there were four LEDs with an arrangement of 90° (see #7 in Figure 3). 

The near-infrared camera of type Goldeye P-008 SWIR Cool from Allied Vision was placed in the center of the oculus of the dome and has a spatial resolution of 320 (H) × 254 (V) pixels (#5 in Figure 3). The camera response wavelength range is from 900 nm to 1700 nm and provides 16-bit digital images with a maximum acquisition of 90 fps. The local resolution for a pixel is 0.23 mm for a picture size of 72.72 mm (H) × 57.66 mm (V). The exposure time of the camera was configured by 0.03125 s. The exposure time is adjustable and indicates how long the light was collected and integrated by a camera. The synchronization between each wavelength light beam and the image acquisition was achieved by using a microcontroller (#2 in Figure 3) and sequenced via a LabVIEW^®^ Program in the computer unit at 20 fps (#1 in Figure 3). 

A syringe pump with two syringes (#8 in Figure 3) injected glycerol and water into the two entrances of the mini-channel (#9 in Figure 3). The mini-channel was placed under the dome lighting unit and in front of the camera system at a distance of 20 cm. The temperature of the mini-channel was regulated by using a heater and a temperature controller (FA-5082-3, HP113-D, FIRST, Austria), with a temperature sensor type K to achieve the required temperature for the measurement (#10, #3, and #4 in Figure 3). 

**Figure 4:**
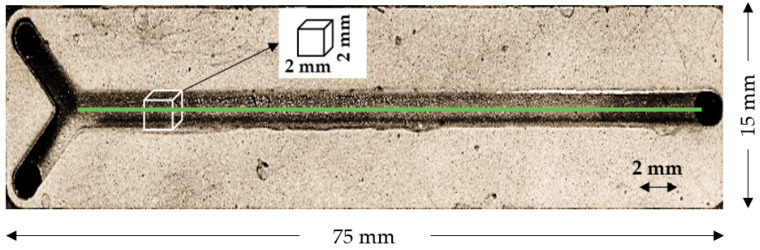
Top-down view of aluminum Y-junction mini-channel with two inlets and one outlet; square cross-section of 2 mm × 2 mm, 75-mm channel length. The line in green is used to calculate the intensity at a wavelength.

The mini-channel used for these experiments is made of aluminum (CN AW 2007—AlCu4PbMgMn) (#9 in Figure 3 and Figure 4). This material is chemical-resistant and suitable for all chemicals used in this experiment. Additionally, with this material, it is convenient to control the temperature of fluids inside the channel by attaching a heating or cooling unit outside due to high thermal conductivity. The mini-channel itself is sandblasted with spherical glass beads (40–70 μm; DIN 52 900; from ARTEKA) to ensure a dull and smooth channel surface for minimal reflection and optimal fluid flow. The dimensions of the mini-channel are 2 mm × 2 mm × 75 mm, with a Y-shaped inlet geometry. 

The Y-shaped inlet geometry is used for the non-turbulent accumulation of fluids next to each other at the beginning of the channel in comparison to a T-shaped inlet. Additionally, with a T-shaped inlet, it is not possible to detect the pure fluids at the beginning of each individual mini-channel inlet branch since the mixing process is already taking place in the branch. Alternatively, it would be possible to increase the range of the individual inlets by a few millimeters, but this option was disregarded due to the Y-shaped inlet being more convenient for the fluid flow. 

The top of the mini-channel is sealed with a quartz window for suitable visibility of the fluids with the measurement system. For a strong bond between the glass and the metal, a special UV-adhesive (Norland Optical Adhesive 61 LOT 415) was used. This adhesive was applied on top of the mini-channel surface and was subsequently cured via UV light to ensure a stable measurement process and a sealed channel. 

The wavelengths of the LEDs were selected according to the spectra of the most used materials, like water, ethanol, and glycerol, and the camera response. In the range of 900 nm to 1700 nm, seven wavelengths (1050, 1200, 1300, 1450, 1500, 1550, and 1650 nm) were selected (LED1050G-03, LED1200S-03, LED1300-03, LED1450-03, ELD-1500-525, ELD-1550-525, ELD-1650-525, Roithner Lasertechnik GmbH, Wien, Austria). 

**Figure 5:**
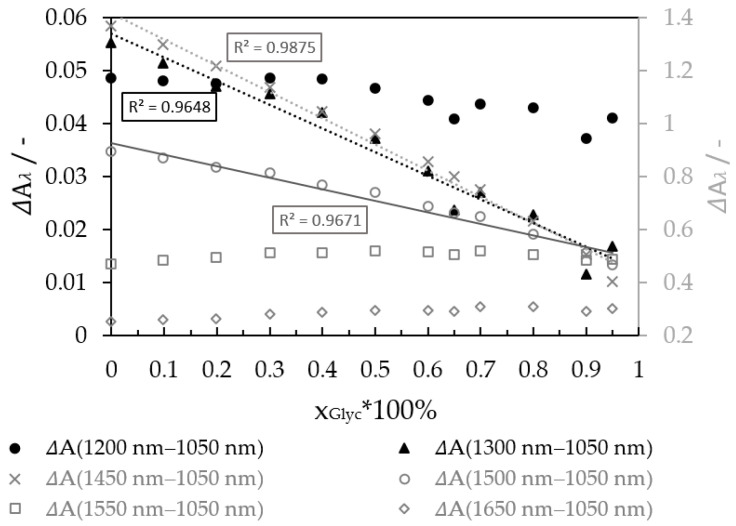
Differences between absorbance by wavelength and the reference 1050 nm.

The spectra of the glycerol-water mixture determined the performance analysis. Through Lambert–Beer law, the concentration, by using the adequate LED wavelength, was calculated. The reference for pure water and pure glycerol is 1050 nm because there is no absorption of light by water and glycerol at this wavelength. Sensitivity for each wavelength pair was calculated to find the suitable wavelength combination and analyze the glycerol-water mixture. Figure 5 shows the calculated absorbance differences by all the integrated wavelengths and the reference 1050 nm. 

Three wavelengths showed a linear behavior dependent on the mass fraction of glycerol.:1300, 1450, and 1500 nm presented the best performance, with a measurement effect in absorbance of 0.038, 0.967, and 0.423, respectively. 

### 2.5. Calibration and Concentration Samples

Determination of the local concentration by mixing two components necessitates calibration. The calibration curves were calculated by evaluating the different concentrations simultaneously and using Equation (11). Thirty images of each of the five glycerol-water mixtures (0, 0.10, 0.50, 0.90, and 0.95 $g_{Glyc}g_{total}^{-1}$) were collected, and the mean intensity for each concentration was calculated. Images obtained from the camera were processed on LabVIEW^®^ and were filtered by using a filter algorithm, whereby failed pixels were detected, eliminated, and averaged to improve the signal-to-noise ratio (SNR).

A pixel calculation was performed to find the suitable wavelength for the analysis. The light intensity of the pixels on a line placed in the middle of the channel was used to determine the performance of the wavelength on the experimental setup (see Figure 4).

**Figure 6:**
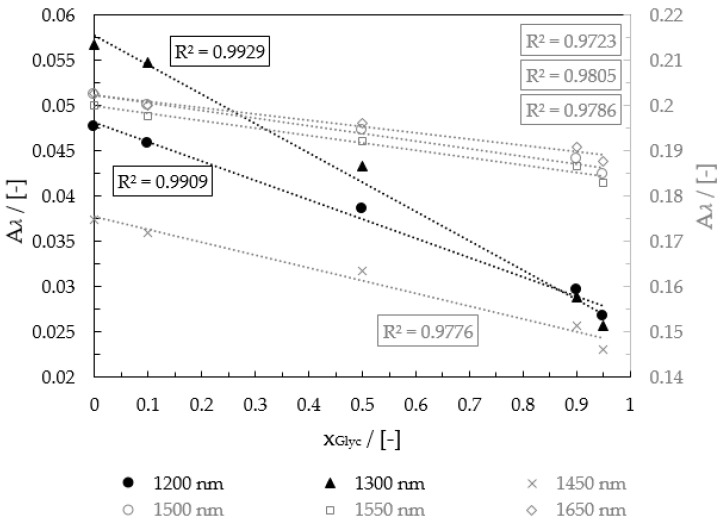
Calibration curves in absorption for a pixel at 20 °C, 30 °C, and 40 °C.

For each wavelength, the absorbance on the trend on the channel was calculated and compared. The absorbance curves of all in the dome-integrated wavelengths at 20 °C are shown in Figure 6. 

In comparison to the analysis with the spectrometer shown in Figure 5, where 1450 and 1500 nm were the adequate wavelengths, 1300 nm had the most suitable performance with linear behavior, a coefficient of determination of 0.9929, and a measurement effect of 0.0311 between 0.05 and 1.00 $g_{Glyc}g_{total}^{-1}$. For this reason, this wavelength was used in this work for the determination of the mass fraction of glycerol-water mixtures. 

**Figure 7:**
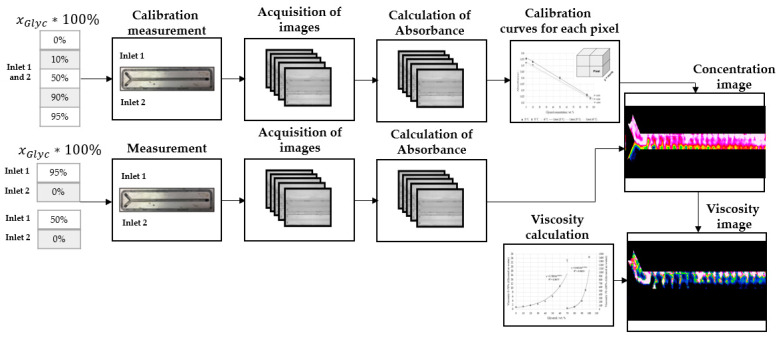
Schematic experimental workflow for the calibration and evaluation of the near-infrared measurement results.

By using pictures taken with 1300 and 1050 nm LED, the absorbance picture was calculated for each concentration. From these, the viscosity maps were deduced. Each pixel of this picture was analyzed separately, and a calibration curve for each pixel was determined. A pixel calibration avoids the adverse effects of lighting conditions from the dome and mini-channel imperfections due to the mounting of the quartz window with the UV adhesive. The software saves each pixel calibration curve and evaluates the measurement by using them. Measurement results are then shown as concentration (mass fraction) pictures. Figure 7 shows the schematic experimental workflow for the processing of the images, calibration, and determination of the concentration.

## 3. Results and Discussion

**Figure 8:**
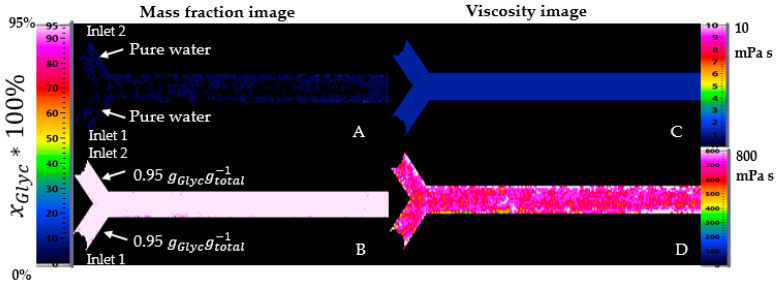
Calibration pictures for pure water (**A**) and glycerol-water mixture with $x_{Glyc}$ = 0.95 $g_{Glyc}g_{total}^{-1}$ (**B**) at 20 °C and their calculated viscosity images (**C**,**D**).

After the calibration, the resulting pictures were saved and used to calibrate the measurement. Figure 8 shows the results for pure water in both inlets (A) and a mixture with $x_{Glyc}$ = 0.95 $g_{Glyc}g_{total}^{-1}$ in both inlets (B). Figure 8C,D are the calculated viscosities of (A) and (B), respectively. Images are shown in false color for a suitable visualization. At 20 °C, the viscosity of water is rounded to 1 mPa s. At the same temperature, a mixture with $x_{Glyc}$ = 0.95 $g_{Glyc}g_{total}^{-1}$ has a viscosity of 515 mPa s.

**Figure 9:**
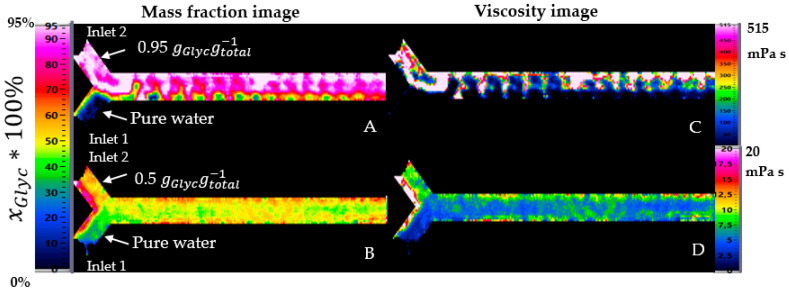
Concentration in mass fraction and viscosity profile in the channel. The viscosity is calculated by using the local concentration in mass fraction and Equation (1). (**A**) local concentration in mass fraction at significant viscosity difference, (**B**) local concentration at small viscosity difference (**C**) calculated local viscosity from A and (**D**) calculated local viscosity from B.

For the proof-of-concept-study, two different cases with a distinct difference were studied. For the first case, an analysis at significant viscosity difference was performed using pure water with a flow rate of 3.3 mL/min in inlet one and glycerol-water mixture with $x_{Glyc}$ = 0.95 $g_{Glyc}g_{total}^{-1}$ with a flow rate of 2 mL/min in inlet two of the mini-channel. Using these two flow rates and at 20 °C, the mass fraction of glycerol was $x_{Glyc}$ = 0.4174 $g_{Glyc}g_{total}^{-1}$ by completed mixing, calculated with the flow rates in each inlet. For the second case, a slight viscosity difference was studied using a mass fraction of glycerol with $x_{Glyc}$ = 0.50 $g_{Glyc}g_{total}^{-1}$ in inlet two with a flow rate of 3 mL/min and pure water in inlet one of the channel at the same flow rate. Here is the mass fraction of glycerol $x_{Glyc}$ = 0.2995 $g_{Glyc}g_{total}^{-1}$ by completed mixing at 20 °C. Figure 9 summarizes the two regimes for the mixing comparison. 

At a large viscosity difference, water flows in the lower part of the channel and mixes slowly with the glycerol. Glycerol takes about two-thirds of the channel and thus presses the water away, and glycerol flows at a high mass fraction on the channel length. Therefore, the mixing was not optimal on the channel. At a small viscosity difference, glycerol and water were better mixed even from the very beginning. There was no clear separation between glycerol at 0.5 $g_{Glyc}g_{total}^{-1}$ in inlet two and pure water in inlet one. Water flows at a high concentration only in the first part of the inlet. After that, the concentration is approximately 0.5 $g_{Glyc}g_{total}^{-1}$, different from the expected concentration of 0.30 $g_{Glyc}g_{total}^{-1}$ by completed mixing at 20 °C.

An indirect calculation of the viscosity is possible by using Equation (1). The values of the dynamic viscosity depend essentially on the concentration and temperature of the mixture. Figure 9 shows the two cases as concentration images and their calculated local viscosity as images. Experiments with a large viscosity difference between input one and input two (Figure 9A) and with a small viscosity difference between the same inputs (Figure 9B) were carried out and explained before. With a significant viscosity difference, a clear transition between water and glycerol is visible.

For the first case, the mass fraction image A is converted in viscosity image (C). By using a maximal mass fraction of 0.95 $g_{Glyc}g_{total}^{-1}$, the maximal calculated viscosity is 516 mPa s. The color scale of the viscosity image was configured with a maximum value of 515 mPa.s for a suitable visualization. For the second case, the mass fraction image (B) was also converted in viscosity image (D), with a maximal viscosity of 8 mPa s. 

### Effect of Temperature

**Figure 10:**
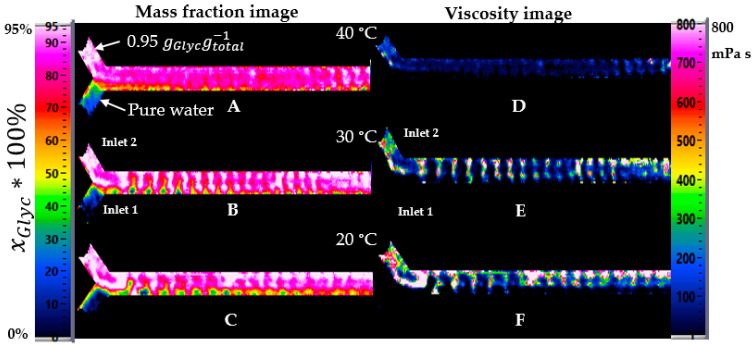
Effect of the temperature on the concentration in mass fraction mapping from channel at 40, 30, and 20 °C (**A**–**C**) for a large difference of viscosity between inlet 1 water and inlet 2 with 0.95 $g_{Glyc}g_{total}^{-1}$. (**D**–**F**) Viscosity mapping from channel at these three temperatures.

The effect of temperature on mixing was analyzed and visualized by three temperatures. A further increment of the temperature from 20 to 30 °C and then to 40 °C shows a different effect in the variation of the local mass fraction in the channel. Figure 10 shows the two regimes with a significant difference in viscosity at the same flow rate, 3.3 mL/min for glycerol-water mixture and 2 mL/min for water. 

With a significant viscosity difference between inlet 1 and 2 (Figure 10A–C), water and glycerol formed a boundary layer on the Y-shape and flowed separately at 20 °C. At 30 °C, water and glycerol began to mix, and at 40 °C, pure water was not visible on the length of the channel.

**Figure 11:**
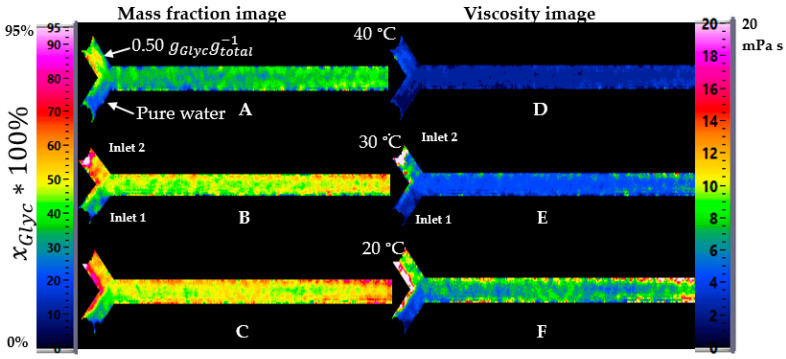
Effect of the temperature on the concentration in mass fraction mapping from channel at 40, 30, and 20 °C (**A**–**C**) for a slight difference of viscosity between inlet 1 water and inlet 2 with 0.50 $g_{Glyc}g_{total}^{-1}$. (**D**–**F**) Viscosity mapping from channel at these three temperatures.

**Figure 12:**
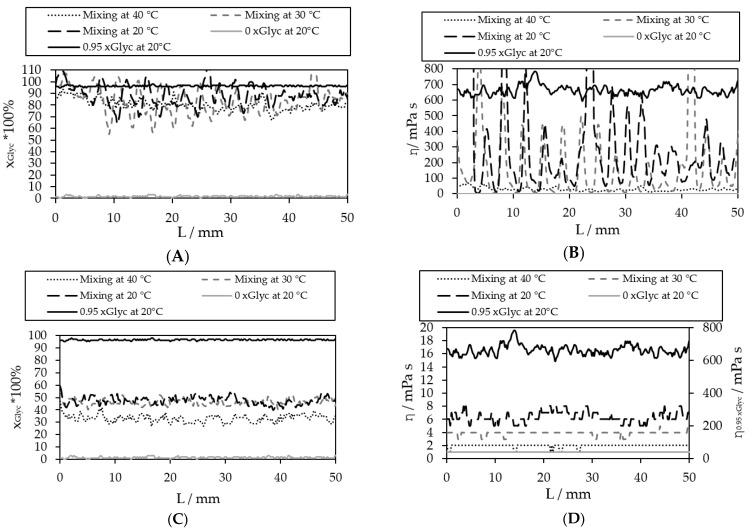
The trend in the channel (as in Figure 9) for the first instance at high viscosity difference (**A**,**B**), for concentration in mass fraction image (**A**) and viscosity (**B**), and slight viscosity difference (**C**,**D**) for concentration $x_{Glyc}$ images (**C**) and viscosity images (**D**).

With minor viscosity differences (Figure 11D–F), water and glycerol had no clear transition on the Y-shape; they mixed immediately, and even at 20 °C, the concentration was stable along the length of the channel. However, the expected concentration was not reached at 20 °C. The trends in the center of the channel for the three temperatures and the two regimes are shown in Figure 12 for improved comparison.

At high viscosity difference between inlet one and two (Figure 12A), a minimal $x_{Glyc}$= 0.70 $g_{Glyc}g_{total}^{-1}$ was reached at 40 °C. It was significantly different from the expected $x_{Glyc}$= 0.42 $g_{Glyc}g_{total}^{-1}$ in the channel by an optimal, completed mixing (calculated with the flow rate in each inlet). At a small viscosity difference at 40 °C (Figure 12C), the expected value was $x_{Glyc}$ = 0.30 $g_{Glyc}g_{total}^{-1}$. At 20 °C and 30 °C, the concentration $x_{Glyc}$ in the channel was between 0.45–0.50 $g_{Glyc}g_{total}^{-1}$, respectively, and at 40 °C, the expected concentration $x_{Glyc}$ was reached. The values in the center of the channel from the calculation of the viscosity images is shown in Figure 12B,D.

## 4. Conclusions

The contributions present a proof-of-concept-study based on an imaging analysis that allows investigating the mixing of widely differing fluids with regard to their viscosity. The experiments were carried out in a mini-channel because of the visualization of the inlets and the possibility of setting the desired parameters by controlling the temperature and the flow ratio. 

The presented near-infrared imaging analysis method for mixing processes in a mini-channel permits the calculations of different mixing parameters, such as local concentration, absorption, and viscosity. The suitable wavelength used for these calculations was found to be 1300 nm and, as reference, wavelength 1050 nm. Around 1300 nm wavelength, extinction of glycerol was low, which allowed the detection of glycerol-water mixtures up to 2 mm. 

The selected fluids were chosen as model substances in this study because of their non-toxicity and ease of handling in the laboratory setting as well as the analysis of large differences in the viscosity. After calibration, the near-infrared image analysis can be used to determine local concentrations simultaneously at each location within the mini-channel. It should be kept in mind that although the presented method has the advantage of simultaneous detection of all local conditions, on the other hand, the nature of the measurement requires integration over the different possible concentrations in depth. The whole methodology is based on the fact that the light penetrates the liquid layer twice, and depending on the molecular composition, different near-infrared wavelengths are absorbed differently. Thus, local concentration is an averaged value in the depth direction at each point of the channel. Local reference measurements in depth were not possible to conduct in this study but are planned for future measures by including other spectral modes, such as Raman or fluorescence spectrometry. 

The advantage of the experimental design is that, with a possible spectroscopic calculation of local concentrations, viscosities can also be derived indirectly. Viscosity differences come from concentration differences but also from changing temperatures. The concentration profiles and mixing processes within the channel can be optically visualized using near-infrared calculation. Thus, it is always possible to draw a viscosity map for an experimental moment under the constraints of assuming homogeneous height profiles. 

The fluctuating flow progressions are a generally known phenomenon. The resulting highly inhomogeneous flow at high-viscosity differences is precisely the difficulty for numerical flow simulations and their fundamental theories. The fluids involved in this case have so far been difficult to capture for numerical flow simulations precisely because of their widely varying viscosities. Then, the motivation of the experimental analysis presented here was to explain the technology and to provide in future works experimental data as a reference for computational fluid dynamics.

With the design presented here, it is now possible to investigate the influences of different feed flows, flow disturbances, and potentially the confluence of three-phase systems in the following work. 

## Figures and Tables

**Table 1:** Dynamic viscosity for the calibration mixtures at 20, 30, and 40 °C.

$\mathrm{Glycerol}\;\mathrm{Mass}\;\mathrm{Fraction}\;(g_{Glyc}g_{total}^{-1})$	Viscosity (mPa s)		
	20 °C	30 °C	40 °C
0.00	1.005	0.799	0.654
0.10	1.297	1.020	0.826
0.50	6.002	4.224	3.111
0.90	223.7	112.0	61.53
0.95	1414	597.9	282.4

**Table 2:** Equations for imaging analysis of viscosity at 20, 30, and 40 °C.

Temperature (°C)	Glycerol Mass Fraction 0–0.7 $g_{Glyc}g_{total}^{-1}$	$R^{2}$	Glycerol Mass Fraction 0.7–1 $g_{Glyc}g_{total}^{-1}$	$R^{2}$
20	$\eta_{mix}=0.7893e^{4.37x_{Glyc}}$	0.9677	$\eta_{mix}=0.0015e^{13.51x_{Glyc}}$	0.9815
30	$\eta_{mix}=0.6466e^{4.03x_{Glyc}}$	0.9704	$\eta_{mix}=0.0023e^{12.24x_{Glyc}}$	0.9812
40	$\eta_{mix}=0.5426e^{3.74x_{Glyc}}$	0.9729	$\eta_{mix}=0.0033e^{11.12x_{Glyc}}$	0.9810

## Data Availability

The data presented in this study are available on request from the corresponding author.

## Appendix A

**Table A1:** Coefficients a and b for the three temperatures.

*ϑ* (°C)	*a*	*b*
20	0.671	2.073
30	0.654	2.068
40	0.637	2.053

**Table A2:** Properties of working fluid at 20 °C. The Reynolds numbers were calculated for a flow of 3mL/min in each inlet and using the calculation of density of glycerol-water mixture at 20 °C.

			Cheng [28]	Segur and Oberstar [23]	P.N.Shankar [25]
Glycerol Mass Fraction $\left(g_{Glyc}g_{total}^{-1}\right)$	Fluid Density (kg m^–3^)	Reynold Number	Viscosity (mPa s)	Viscosity (mPa s)	Viscosity (mPa s)
0	998.0	49.66	1.005	1.005	0.984
0.1	1024	39.49	1.297	1.310	1.274
0.2	1051	30.18	1.741	1.760	1.734
0.3	1078	21.95	2.455	2.500	2.462
0.4	1104	14.96	3.685	3.720	3.728
0.5	1131	9.421	6.002	6.000	5.886
0.6	1158	5.304	10.91	10.80	10.53
0.7	1184	2.564	23.09	22.50	21.89
0.8	1211	0.995	60.86	60.10	55.84
0.9	1237	0.277	223.7	219.0	229.7
0.95	1251	0.121	515.6	523.0	232.4
1	1264	0.045	1413	1412	1466
